# Information Experiences and Needs in Patients with Pulmonary Arterial Hypertension or Chronic Thromboembolic Pulmonary Hypertension

**DOI:** 10.1155/2014/704094

**Published:** 2014-08-17

**Authors:** Bodil Ivarsson, Björn Ekmehag, Trygve Sjöberg

**Affiliations:** ^1^Department of Cardiothoracic Surgery, Lund University and Skåne University Hospital, 221 85 Lund, Sweden; ^2^Medical Services, 221 85 Lund, Sweden; ^3^Uppsala University Hospital, 751 85 Uppsala, Sweden

## Abstract

*Background*. Pulmonary arterial hypertension (PAH) and chronic thromboembolic pulmonary hypertension (CTEPH) are fatal, noncurable, but treatable diseases that strongly affect the patients. *Objective*. To describe patients' experience of information relating to PAH or CTEPH. *Methods*. A qualitative method using content analysis was applied. Seventeen patients (thirteen women and four men) aged 28–73 years from a regional PAH centre were individually interviewed. *Results*. Three categories that describe patients' experiences of information emerged: handling of information, struggling with feelings that also affect others, and vulnerability associated with uncertainty. The patients would have welcomed more information to relatives from the healthcare professionals. Shortcomings on communicating a prognosis were experienced. The mediated information and knowledge gave the patients insight into physical or psychosocial problems. Mutual exchange of information between patients and healthcare professionals were marred by different experiences of attitudes, behaviour, and ownership. *Conclusions*. In the future, healthcare organizations must struggle to achieve a holistic healthcare by making it more person-centred, and they must also promote cooperation between PAH centres and local healthcare providers. It is essential to determine the most appropriate and valuable path of information and communication and, thereby, the most cost-effective management of PAH or CTEPH.

## 1. Introduction

Pulmonary arterial hypertension (PAH) and chronic thromboembolic pulmonary hypertension (CTEPH), both part of different groups of pulmonary hypertension (PH), are fatal, rare diseases, with an overall prevalence of 15–50 individuals per million for PAH, whereas the incidence of CTEPH is more uncertain [1, 2]. In 2013, in Sweden, roughly 590 patients are affected by PH and the mean age at diagnosis is 54 years for women and 58 for men. Nearly twice as many women as men are affected [3].

PAH is an umbrella term for a group of diseases that affect the pulmonary vascular bed, leading to an increased workload for the heart's right ventricle and, with time, often chronic heart failure [4]. Patients with PAH face a severe rapidly progressive condition that without treatment often leads to death within a short period of time [5]. According to historic registry data [6] of patients between 1981 and 1988, the median survival without modern treatment was 2.8 years, one-year survival was 68%, and 5-year survival was only 34%. At the end of 2013 Swedish registry data of all PH patients showed that one-, three-, and five-year survival were 89%, 75%, and 65%, respectively [3]. The knowledge about PAH and CTEPH, both of which are handled according to the same specific pharmacological treatment and same investigation scheme, has increased rapidly during the last 20 years [1]. A number of pharmacological treatment options, for example, endothelin receptor antagonists (ERAs), 5-phosphodiesterase inhibitors (PDEi), and prostacyclin analogues (PRO), are now available which can improve quality of life as well as exercise capacity [1]. There is also plausible evidence that modern treatment has had a significant and positive effect on survival [7]. However, the symptoms in the initial phase are often diffuse and uncharacteristic; often patients are inexplicably tired and suffer from dyspnea [8]. Many patients have met experienced specialists in healthcare over an extended period of time during which the disease has not been identified and therefore a correct diagnosis and relevant treatment have been delayed [9, 10]. This is a common situation worldwide due to insufficient knowledge of PAH and CTEPH among healthcare professionals [11]. Given the complex nature of the disease, and in order to achieve the best outcomes of the treatment of PAH and CTEPH, it is important that patients be treated by a multidisciplinary specialist team consisting of physicians, nurses, physiotherapists, social workers, and so forth, with extensive experience in PAH [1, 2]. Furthermore, the multidisciplinary specialist team must have the knowledge and resources to diagnose, treat, support, and provide information to patients and their families in order to optimise the conditions for a good quality of life, despite the chronic illness [10].

Life as a patient with PAH or CTEPH, and thereby chronic illness, includes avoiding harmful situations, taking medications, and having regular contact with healthcare organisations [8, 12]. It is of utmost importance that healthcare professionals recognize their key role as informers and it is essential to ensure that patients understand and follow the advice they receive regarding medication, diet, and exercise [12]. According to Sanford [13], a caring context must be characterised by relationship, dialogue, mutual trust, shared meaning, and receptivity. The healthcare professionals must have a patient-centered approach, in which the patients are encouraged to participate and in which the professionals make sure they have understood the information and clarify if there are questions [14]. In addition, the caring context requires well-designed measures that actively engage patients in their own care, thereby improving their health [15].

In this study we relate information, communication, and patient education; however in the text the concept information is used. To date, several authors outline the benefits of providing PAH and CTEPH patients with information [16, 17] but there has been a lack of empirical studies regarding information. Therefore, the overall aim of this study was to describe patients' experiences and needs of information relating to PAH and CTEPH.

## 2. Methods

### 2.1. Design and Patients

A qualitative, descriptive approach was used for the study. Seventeen patients, diagnosed with PAH or CTEPH, from one university-affiliated PAH centre in Sweden were strategically chosen in order to achieve variations in patient experiences and in sociodemographic data such as sex, age, diagnosis, and marital and domicile status [18]. Demographic data and characteristics of the patients are given in Table 1.

### 2.2. Ethical Considerations

The investigation conforms with the principles outlined in the Declaration of Helsinki [19]. The Regional Ethical Review Board in NN, Sweden, approved the study (LU 2011/364). Permission to contact informants was given by the director of the PAH centre. All the participants were given written and oral information about the study and its purpose, and all were informed that they could withdraw at any time without explanation or consequence. Furthermore, they were assured that the material would be treated as strictly confidential. All participants signed a written consent letter. So as not to abandon the patients with their thoughts after interviews, the patients were given the opportunity to contact a social worker.

### 2.3. Data Collection

The patients were later contacted by phone and asked whether they agreed to participate and, if so, where they preferred the interview to take place. The face-to-face interviews were semistructured and covered the following topics: (a) demographic details such as education and marital status and (b) questions about information and support. The introductory question was “Please tell me about your experience and needs of information, communication or education in connection with PAH or CTEPH.” A question about their experience of support in connection with PAH or CTEPH was also asked (the result is reported elsewhere). Follow-up questions were asked in order to clarify the narratives and to continue the conversation. In order to secure the content and quality of the interview, the first interview served as a pilot test. It was deemed satisfactory and could therefore be included in the study. In the last three interviews no new concepts emerged, which indicated that a sufficient number of interviews had been conducted. The interviews, conducted between January and October 2012, lasted between 19 and 67 minutes and were conducted either in the home of the patient (10) or at a neutral office in some healthcare facility (7). The interviews were undertaken in a dialogue form and were digitally recorded. A verbatim transcription of each interview was later made. The patients had no prior relation to the interviewer (BI).

### 2.4. Data Analysis

The text was analysed stepwise by means of qualitative content analyses as proposed by Burnard [20]. In the first step, all text was repeatedly read through by two of the researchers (BI and TS) so as to provide an overview. In the second step, the text was again carefully read, focusing on identifying meaning units that were related to the aim of the study. In step three, the meaning units were condensed and labelled with codes and sorted into 10 subcategories that met the aim of the study. In the final step, to ensure reliability, one researcher (TS) made a detailed examination of the first researcher's (BI) coding and categorisation and adjustments were made until satisfactory agreement was established. Thereafter, three overall categories were developed [21]. The result has been supported with quotations so as to enable the reader to judge the trustworthiness.

## 3. Result

Patients' experience of information is presented in three categories and 10 subcategories (Table 2).

### 3.1. Handling of Information

This category describes how the patients experienced the information they received, or missed, regarding PAH or CTEPH.

#### 3.1.1. Winding Road to a PAH Specialist Care Team

Most patients described how they repeatedly sought care at medical services in primary care, emergency departments, and specialist clinics during a long period of time. They described that they underwent different investigations and received information about a wide range of diagnoses before they finally came into contact with the PAH team. Then, they gained insight and received professional information, care, and treatment. However, they felt that it was sometimes difficult to make contact with the PAH team between scheduled visits, especially the physicians. The patients wished that they could be continuously updated from the healthcare professionals with detailed information as to what was happening in the PAH or CTEPH field and the reason for different physiological tests.

*The PAH-team; they seem to be very competent. All of them know what they're doing and saying, they're friendly and nice … they even do research about it. It's fantastic. (P 1)*

#### 3.1.2. Usefulness of Written Information

Some remember that they received a leaflet about PAH and CTEPH which was mostly general. These were satisfied with the written information to supplement the oral information, and that they could read this in peace and quiet at home and even show it to their relatives. The patients wished to have a written overview with names of the physician and nurse in charge, including telephone numbers, their own medication list, and blood test results, as a support for their memory, but to also show other healthcare providers.

*At first, when I read the folder I thought, no, I'm not this sick, but obviously I was. It's just the way it is, so whoever's written it knows what they're doing. (P 15)*

#### 3.1.3. Lacking Information to Relatives

Only a few patients said that relatives received information on PAH or CTEPH from healthcare professionals and if so, it had occurred randomly. Most patients felt that they had to inform relatives themselves and they found it difficult to give proper information about the illness and its treatment. Patients wished that relatives had received some of the information from healthcare professionals. That would increase the relatives' understanding of the disease and should make it easier for the patient to remember everything that had been said in the discussions with healthcare professionals. Some patients felt that the family did not have insight or understand the meaning of being diagnosed with PAH or CTEPH, while others felt that if the relatives did not receive information from healthcare professionals they would be looking on the internet.

*She [my wife] has more questions than I do and she's never been given any information directly or been able to be present at the meetings. No-one's actually denied her but then again, no-one has ever said that it would be very good if your wife were here with you. (P 3)*

#### 3.1.4. Using and Avoiding Internet as a Source of Information and Knowledge

Most patients had sought information about PAH and CTEPH on the internet. It was through this information that they understood that PAH and CTEPH were serious and that, in order to increase survival time, they would have to take medication for the rest of their lives. They recognized that Swedish specialist physicians responded to questions in a specific web-based forum for PAH patients. They felt that this site had high quality; otherwise they were very critical to the source of information on the internet, but occasionally they used the information they found to formulate questions to the PAH team.

*I read what I found on the Internet but that was too difficult, all I could focus on there was that it's a very serious disease. Then I panicked … so, when I look back, I realise I felt like I was really at the mercy of a strange diagnosis that no-one knew anything about. (P 6)*

#### 3.1.5. The Unspoken about the Prognosis

The patients had questions about their prognosis. They tried to get information from the physicians about their situation and about what was correct in terms of their own prognosis, but that was difficult to achieve. The patients thought that this was because the physicians knew that new drugs were under development and that was why they had difficulties with prognostication. The patients expressed satisfaction with knowing that they would be called to frequent follow-ups and this strengthened their trust and hope. Some patients felt so good after they received medication that they, at times, almost questioned if they really had the disease when they compared themselves with others with PAH or CTEPH.

*I wasn't alarmed … I'll fix this, I'm stubborn. I can get help from medicines, because it was really, really bad. (P 5)*

#### 3.1.6. Being Rational regarding Surgical Procedures

Patients with CTEPH mentioned that for some thromboendarterectomy is an alternative and some had undergone this. The surgery in these cases was not performed at the hospital where the PAH center was located and the patients stated that they, to a great extent, had to organize everything themselves and that most of the information they received was from a remote medical team. They had wanted more collaboration between PAH center and surgical hospital. Most patients reported that they knew that a lung transplant was the ultimate chance for survival. Some described that they received information about lung transplantation from the PAH team and thereafter realized that thoughts about a life without medication, about getting pregnant, and about the fatigue disappearing were not realistic.

*Now, afterwards, I'm glad that I'm not there yet with a transplantation; almost thought it seemed better than being on life-long medication; I wasn't in my right mind. (P 7)*

### 3.2. Struggling with Feelings That Also Affect Others

This category describes information leading to insight of physical or psychosocial burdens experienced by patients because of their illness.

#### 3.2.1. Fear of Hereditary Disease

Many patients had concerns about PAH or CTEPH and heredity. They had discussed their concerns with the specialists and were informed that there were no indications that their PAH or CTEPH was hereditary. Despite this, they could not get rid of their fear. Some patients had parents who had died at a young age and they now had concerns that the reason was PAH. Many thought of their children, and some had very carefully informed their children to be observant for signs of the disease, largely because of their own experiences of the long and uncertain duration before getting diagnosed and receiving treatment by PAH specialists.

*…thought, what'll happen now with my child, is it hereditary and when my child said he was too tired to do gym I got so scared and thought that was a sign. How early on can you notice it? (P 7)*

#### 3.2.2. Feelings of Injustice in Not Being Able to Have Children

Fertile women expressed feelings of sadness due to not being able to give birth. Some had had experiences of abortion because of their PAH. They described that they did not even qualify to adopt a child, which requires a health certificate. They felt that the question of children also greatly affected their partners as well as other members of the family. Their experience was that the information provided by the PAH team was straight and honest, and they trusted their knowledge. However, these patients lacked someone in the healthcare system with whom to exchange thoughts on this topic. Patients of both sexes had a lot of thoughts about children, about the amount of energy required to take care of children, and thus about their own caring ability.

*The doctors said that it's not a good idea getting pregnant because I've got PAH. It can work out but it can also go really wrong. You need a lot of energy for children and he's so right! So he said I should be satisfied with the child I already have, and I am. (P 17)*

### 3.3. Vulnerability Associated with Uncertainty

This category describes experiences of attitudes, behaviour, and ownership.

#### 3.3.1. Failing Healthcare Organization

Most patients felt that their physicians at remote healthcare centres did not have enough knowledge of PAH and CTEPH. Their experience was that they often had to provide information about both the disease and the drugs on their visits to health centres. The patients requested more contact between the PAH team and remote healthcare centres. If they needed an ambulance when visiting emergency or other clinics, they often felt that they had to be emphatic in saying how sick they were. Some patients even pleaded to the healthcare professionals to increase their knowledge of PAH and CTEPH as the staff did not realize what was wrong with the patient. Patients expressed their wish that the caregivers would respect the fact, to a greater extent, that they themselves had the most comprehensive knowledge of their own condition. Some meant that they should wear a card showing that they suffered from PAH or CTEPH which explained specific tasks and symptoms regarding PAH or CTEPH.

*If the infection found its way down into my lungs I'd get in touch with the healthcare centre or the emergency unit and they'd send me home. I mean the level of knowledge is really quite low, you cannot always see there's anything wrong by just looking at me. You can look in the records, and see that I've had the same problem before and then they'd ask me—how do you know? You can feel it coming. (P 3)*

#### 3.3.2. Pondering about Why They Are Affected

Most patients had their own idea of why they developed PAH or CTEPH. In most cases these ideas were unconfirmed by healthcare professionals, for example, physicians. Examples of unconfirmed causes were smoking, having worked with thinner and asbestos, external violence, severe colds, sepsis, hereditary, tick bites, or some higher power. Exceptions to these reactions involved patients with drug abuse, drug reactions, and systemic sclerosis, where the cause was more obvious. Some blamed themselves for being affected by PAH. There was a particular need to know and some patients reported that they tested their theories when they met a new doctor.

*It seems like I've acquired it in some way. Yes, I have worked with asbestos and I have sat passively with smokers … but, I do not know, they haven't approved, I've tried to get it classified as a working injury … Yes, of course I do think about it from time to time…. (P 4)*

## 4. Discussion

The present study showed that a diagnosis of PAH and CTEPH is associated with a great need for information, specially tailored to the patients' individual requirements and conditions.

After the onset of PAH or CTEPH symptoms, patients in this study often experienced misdiagnosis and delay, sometimes for years up until they received a correct diagnosis and this delay caused reduced benefit of treatment, which should be started as early as possible [17, 22]. This demonstrates the need for educational resources and training programmes in order to increase knowledge about PAH and CTEPH to establish routines for appropriately referring patients to skilled centres for further assessment and possible treatment.

The patients in this study felt that they had received proper oral information by the PAH team. The written information they received did not fulfill their needs but the patients had suggestions for what proper information could contain: all patients should be given the opportunity to get objective, patient-oriented, up-to-date, reliable, understandable, and accessible oral and written information as well as opportunities to discuss their situation as a whole with healthcare professionals. Healthcare professionals should perhaps, to a greater degree, begin by listening to the patients and by documenting patient narratives as described in the person-centered care concept [23].

The findings reveal that several patients reported that relatives were not invited by the healthcare professionals to get information when patients had a confirmed PAH or CTEPH diagnosis. The patients would have welcomed an initiative from the healthcare professionals, especially when they needed support from relatives and when there was a need to increase understanding from relatives. The healthcare organization should be better prepared and should take care of the contact with relatives, who largely account for the emotional support, involvement in medical care, and self-management [24].

According to earlier studies, Internet was the place where the patients found information about the fatal outcome of PAH or CTEPH disease [16, 17]. Patients in this study preferred the PAH teams' more nuanced and more personalized information in contrast to the Internet. One solution is to set up patient-educational interactive web sites, with easy access to specialists physician, but the patients also need access to other categories of the PAH team members, such as experienced nurses or social workers. On one hand, patients in this study requested information about their prognosis and would like the physician to bring up this question. On the other hand, patients felt confidence in that the physicians expressed vague and unclear prognosis information and did not mention the possibility of death due to PAH or CTEPH. This ambiguity may partly be explained by the effects new treatments have in many cases, resulting in an improved level of function and physical performance of patients, as well as significantly improved prognoses. Despite this paradox and despite the fact that the effects of new pharmacological therapy have improved markedly over the last years, a goal for the healthcare professionals should be that the patients do not receive conflicting information about their prognosis. Anderson et al. [25] recommend physician communication with patients about prognosis by starting with the question “What is your understanding of your illness?” Furthermore, all patients should be given the opportunity to discuss goals, hopes, fears, and thoughts about the end of life [25].

Most of the patients know that lung- or heart-lung transplantation may be an alternative treatment option for a selected group of patients when pharmacological treatment fails, but when they heard it for the first time they did not understand that it was a last option of treatment. However, one study has shown that more than half of PAH patients show signs of cognitive impairment [26] which could affect their ability to assimilate information. Therefore it is particularly important that the physicians explore the patients understanding of the illness and information [25].

A review by Bédard et al. [27] showed that much emphasis is put on the prevention of pregnancy. It was stated that if it is not possible to prevent, the pregnancy should be carefully monitored. It was highlighted that the PAH team, responsible for the care of women with PAH, have the difficult task of preventing their young female patients from becoming pregnant, due to the exceedingly high mortality [27]. Sometimes the physician is also faced with the difficult task of recommending abortion [28]. The women in the present study stated that the information from their physician on why it was not advisable to become pregnant and undergo childbirth was adequate. However, they lacked contact for extended information, discussion, and support for themselves and their partners. This form of supportive care should not be overlooked and perhaps the contact should be with a social worker or psychologist at the designated PAH centre. Gin-Sing [10] has suggested that in the long term some support could also be given by local healthcare providers in close relationship with the PAH centre.

Some patients in the present study, especially those with smoking and drug abuse in their past, felt responsible for, or guilty of, causing their illness. As a healthcare professional it is important not to stigmatize this subgroup in order to reduce the risk of not facilitating patient contact with the healthcare, a necessary requirement for improving quality of life [29].

Although the patients' physician in this study informed that their PAH or CTEPH was not heritable, patients could not stop thinking about this because they knew that PAH may be heritable. These concerns are understandable when familiar/hereditary PAH exist and intensive research on heredity and pathogenesis are in progress [30]. Jones & Clayton [31] stated in a study of PAH patients that genetic testing may be useful, regardless of the result, if high levels of stress were experienced and perhaps this is one way of reducing patients' concerns about themselves and their children.

An absence of coordinated healthcare systems, including sufficient routines for information exchange and dialogue between the PAH team and other healthcare providers, has been identified as a main problem by the patients in this study. Establishing clinical nurse specialists at PAH centres may be a way to support both patients and nurses and others in the patient's surrounding [10, 32]. Clinical nurse specialists can improve their own professional practice and achieve excellence in healthcare in collaboration with physicians and others in PAH teams, to support patients with special healthcare needs, their families, and even other healthcare professionals in different positions.

*Methodological Considerations*. Qualitative content analysis was a suitable method for this study, due to the aim to interpret the content of the data through a systematic process and to find variations in the patients' experiences [33]. Trustworthiness in qualitative studies could be evaluated in terms of credibility, conformability, transferability, and authenticity [34]. To ensure that the research findings, for example, credibility, are consistent in this study with the patient's reality, two researchers worked actively with the analysis. Conformability was strengthened by high objectivity of the researchers because the sole motive of the study was improvement of patient care for patients in this group of illness. Regarding transferability it is the reader who determines the potential for generalization of this study [21]. The main limitations of the study are its retrospective nature, a relatively small sample size, and the fact that it was carried out in a single centre, which affects the generalization potential. Strategic sampling has been used to capture different experiences of patients in order to achieve authenticity. The overrepresentation of women may affect the findings but it reflects the predominance in the patient group.

## 5. Conclusion

The result indicated that the patients would have welcomed a healthcare system that better overarches the gap between PH specialist medical care and other health services. The results also showed that mediated information and knowledge gave the patients insight into physical or psychosocial problems and the patients requested more information to relatives. In the future, healthcare organizations must struggle in order to achieve a holistic healthcare by making it more person-centered and by promoting cooperation between PAH centres and local healthcare providers. It is essential to determine the most appropriate and valuable information and communication path and, thereby, the most cost-effective management of PAH and CTEPH. Future studies, particularly multicentre studies, are needed in order to determine the value of the findings in this study.

## Figures and Tables

**Table 1:** Demographic data and disease characteristics of the patients (*N* = 17).

Gender	13 women/4 men
Age	
Mean ± SD	56 ± 15
Median (range)	60 (28–73)
Years since diagnosis	
Mean ± SD	5 ± 3
Median (range)	4 (1–12)
Marital status	
Single (*n*)	5
Married/living together (*n*)	12
Education	
Elementary school (*n*)	4
High school (*n*)	6
College/university (*n*)	7
Diagnosis	
IPAH^1^ (*n*)	6
APAH^2^ (*n*)	1
IPAH + APAH (*n*)	1
APAH + SSC^3^ (*n*)	4
CTEPH^4^ (*n*)	3
CTEPH with surgery (*n*)	2
Drugs	
ERA^a^ + PDE^b^ + warfarin	7
ERA + PDE	4
ERA	1
ERA + Warfarin	1
ERA + CCB^c^ + warfarin	1
CCB	1
PDE	1
PDE + warfarin	1
Current occupation	
Full time job (*n*)	3
Part time job (*n*)	5
Disability/retirement pension	9

^1^Idiopathic pulmonary arterial hypertension.

^2^Associated pulmonary arterial hypertension.

^3^Sclerosis-associated pulmonary arterial hypertension.

^4^Chronic thromboembolic pulmonary hypertension.

^a^Endothelin receptor antagonists.

^b^Phosphodiesterase.

^c^Calcium channel blockers.

**Table 2:** A summary of categories and subcategories.

Categories	Subcategories
Handling of information	Winding road to a PAH specialist care team
	Usefulness of written information
	Lacking information to relatives
	Using and avoiding Internet as a source of information and knowledge
	The unspoken about the prognosis
	Being rational regarding surgical procedures

Struggling with feelings that also affect others	Fear of hereditary disease
	Feelings of injustice in not being able to have children

Vulnerability associated with uncertainty	Failing healthcare organization
	Pondering about why they are affected
